# Effect of aerobic exercise of different intensity on articular cartilage metabolism in patients with knee osteoarthritis: A randomized controlled trial

**DOI:** 10.1097/MD.0000000000048913

**Published:** 2026-05-29

**Authors:** Liangliang Song, Yuanyuan Meng

**Affiliations:** a Shijiazhuang People’s Hospital, Shijiazhuang, Hebei, China.

**Keywords:** articular, biomarkers, cartilage, exercise therapy, heart rate, knee, magnetic resonance imaging, osteoarthritis, randomized controlled trial

## Abstract

**Background:**

Osteoarthritis (OA) of the knee is a leading cause of disability worldwide, yet optimal exercise intensity for cartilage protection remains unclear. This randomized controlled trial investigated dose–response effects of aerobic exercise intensity on cartilage metabolism, clinical symptoms, and structural outcomes in knee OA patients.

**Methods:**

One hundred twenty participants with knee OA (Kellgren–Lawrence grade 2–3) were randomly assigned to low-intensity (50–60% heart rate reserve [HRR]), moderate-intensity (60–70% HRR), high-intensity (70–80% HRR) treadmill walking or stationary cycling, or a control group for 12 weeks (3 sessions/week). Heart rate was continuously monitored using Polar H10 chest-strap monitors. Primary outcomes were serum C-terminal telopeptide of type II collagen and cartilage oligomeric matrix protein levels. Secondary outcomes included Western Ontario and McMaster Universities Osteoarthritis Index (WOMAC) scores, Visual Analog Scale (VAS) pain, 6-minute walk test, and magnetic resonance imaging-assessed cartilage morphology. Data were analyzed using mixed-model repeated measures analysis of variance with IBM SPSS Statistics version 26.0 (IBM Corp.).

**Results:**

The high-intensity group demonstrated the greatest reductions in C-terminal telopeptide of type II collagen (−28.5%, *P* < .001) and cartilage oligomeric matrix protein (−22.3%, *P* < .001), the largest improvements in WOMAC scores (−45.3%, *P* < .001) and VAS pain (−52.5%, *P* < .001), and the greatest increases in cartilage thickness (+4.2%) and volume (+3.8%). A significant dose–response relationship was confirmed (group × time interaction: *F* (3108) = 4.82, *P* = .003). Among participants in the high-intensity group, 73% achieved the minimal clinically important differences (MCID) for WOMAC total score and 80% achieved the MCID for VAS pain. The 6-minute walk test improvement in the high-intensity group (71 m) exceeded the established MCID of 26 m. No serious adverse events were reported; minor events (transient knee pain exacerbation and delayed-onset muscle soreness) resolved spontaneously within 48 to 72 hours.

**Conclusions:**

Appropriately prescribed high-intensity aerobic exercise safely and effectively improved cartilage metabolism, alleviated symptoms, and enhanced functional capacity in knee OA patients. These findings support revising current conservative exercise recommendations upward when tolerable.

## 1. Introduction

Osteoarthritis (OA) is a degenerative joint disease that significantly impacts global health, with knee OA being particularly prevalent and debilitating.^[1]^ As the world’s population ages and obesity rates rise, the incidence of knee OA is projected to increase substantially, posing a growing challenge to healthcare systems worldwide.^[2,3]^ The pathogenesis of OA is complex, involving many factors contributing to progressive degrading of articular cartilage, remodeling of subchondral bone, and inflammation of the synovium. At the root of both the development and progression of OA is disruption in cartilage metabolism, as biomechanical factors play a central role in this process.^[4]^ In a normal joint, there is a fine balance between the synthesis and breakdown of the components forming the cartilage extracellular matrix. Nonetheless, in the context of OA, this balance is disturbed, resulting in an overall reduction of cartilage tissue.^[5]^ Recent studies have identified a number of biomarkers useful in demonstrating such metabolic changes, among which special attention has been given to C-terminal telopeptide of type II collagen (CTX-II) and cartilage oligomeric matrix protein (COMP) as biomarkers reflecting the degradation and turnover of cartilage respectively.^[6]^ While pharmacological interventions remain the cornerstone in the management of OA, there has been increasing emphasis on non-pharmacological approaches, including exercise therapy. Aerobic exercise has shown promise in alleviating OA symptoms and potentially modifying disease progression.^[7,8]^ However, the optimal intensity of aerobic exercise for managing knee OA remains a subject of debate. Some studies reported that moderate-intensity exercises may exert an optimal effect on pain and functional performance,^[9]^ while other studies have suggested that high-intensity interval training may offer superior benefits with respect to cardiovascular health and possibly cartilage metabolism,^[10]^ though rigorous evidence from completed trials remains limited.

The global burden of knee OA is substantial, with rising rates of total knee arthroplasty reflecting end-stage disease prevalence^[11,12]^ and projections indicating continued increases in arthroplasty demand across multiple countries.^[13,14]^ Complications such as periprosthetic joint infection remain a significant postoperative concern,^[15,16]^ occupational ergonomic exposures contribute to disease burden,^[17]^ and evolving surgical techniques reflect ongoing efforts to optimize arthroplasty outcomes.^[18]^ Musculoskeletal conditions including OA increase the risk of comorbid chronic diseases,^[3]^ and the economic impact extends well beyond direct healthcare costs.^[2]^ These alarming trends underscore the urgent need for effective conservative interventions, particularly exercise-based approaches, that may delay or prevent the need for surgical intervention.^[19–21]^

The pathways by which aerobic exercise influences cartilage metabolism in OA are not fully explained. It has been suggested that the mechanical loading that is imparted by exercise can improve the function of chondrocytes and increase matrix production while simultaneously acting on inflammatory pathways in the joint.^[22,23]^ Improvements in muscle strength and joint stability from exercise can give rise to improved biomechanics, which might reduce loading within the articular cartilage.^[24]^

Given both the potential for exercise to affect cartilage metabolism as well as the uncertainty about the intensity at which this may best be achieved, investigation of the differential effects of various aerobic exercise intensities on cartilage metabolism was warranted in persons with knee OA. This study aimed to fill this knowledge deficit by investigating the effects of low-, moderate-, and high-intensity aerobic exercises on biomarkers reflecting cartilage metabolism, clinical symptoms, and functional outcomes in patients with knee OA. The results of this study were expected to contribute to the design of more specific and efficient exercise programs in knee OA management and have implications for disease modification.

## 2. Materials and methods

### 2.1. Study subjects

For this study, a total of 120 participants with knee OA were enrolled from orthopedic clinics and community health centers. Inclusion was based on adults aged 50 to 75 years who had a clinical and radiographic diagnosis of knee OA by the American College of Rheumatology criteria. The inclusion criteria included a minimum score of 3 out of 10 on the Visual Analog Scale (VAS) and a radiographic Kellgren–Lawrence grade of 2 or 3 in the knees. Exclusion criteria included knee injury or surgery within the last 6 months, any inflammatory arthritis, concomitant conditions representing a contraindication to exercise, cognitive impairment, and regular participation in organized exercise programs within the last 3 months. Patients who were on analgesic medication were allowed to continue their routine dosages throughout the period of study. The sample size calculation was performed using G*Power 3.1 software (Heinrich-Heine-Universität Düsseldorf) based on a mixed-model repeated measures analysis of variance (ANOVA) (4 groups × 3 time points), with a medium effect size *F* = 0.25, power 80%, 5% significance level, and a correlation among repeated measures of 0.5, yielding a required total sample size of 100. Further, sampling was extended by 15% to account for expected dropouts, resulting in a target of 120 participants (30 per group). Written informed consent was obtained from all participants, and ethics approval for the study protocol was obtained from the Institutional Review Board (Approval No. 2025-021). This trial was not prospectively registered in a clinical trial registry. Participants were randomly allocated to one of the following 4 groups: low-intensity, moderate-intensity, high-intensity aerobic exercise, or a control group. The randomization was performed using a computer-generated randomization sequence, with stratification by age and sex to ensure balanced groups. Allocation concealment was ensured using sequentially numbered, opaque, sealed envelopes prepared by an independent statistician not involved in participant enrollment.

### 2.2. Experimental design

This study was conducted as a randomized controlled trial to determine the effects of different aerobic exercise intensities on cartilage metabolism in patients with knee OA.

**Figure 1.**
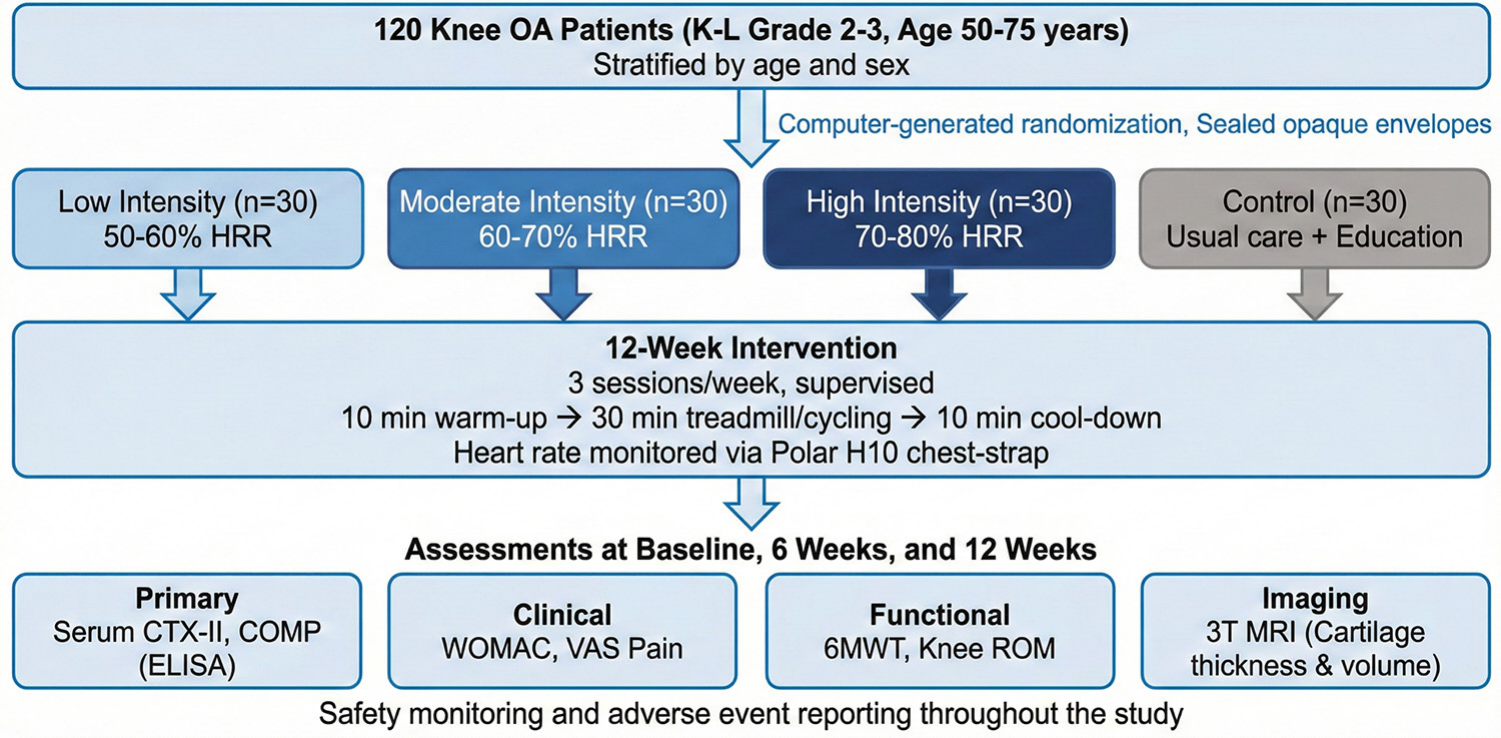
Experimental design framework. Overview of the study protocol including group allocation, intervention schedule, and outcome assessment time points.

Participants were stratified and randomly assigned into 4 groups: low-intensity, moderate-intensity, high-intensity aerobic exercise, or a control group. The intervention lasted for 12 weeks with 3 supervised exercise sessions per week. The low-intensity group performed aerobic exercise at 50 to 60% of heart rate reserve (HRR), the moderate-intensity group at 60 to 70% HRR, and the high-intensity group at 70 to 80% HRR. Exercise intensity was prescribed using the Karvonen formula^[25]^ to calculate target HRR zones. Heart rate was continuously monitored during all exercise sessions using Polar H10 chest-strap heart rate monitors (Polar Electro Oy), with data recorded at 1-second intervals via the Polar Beat application. Each session included a 10-minute warm-up followed by 30 minutes of aerobic exercise, either treadmill walking or stationary cycling (participants selected their preferred modality, which was maintained throughout the study), followed by a 10-minute cool-down. The distribution of exercise modality was similar across groups (treadmill/cycling: low-intensity 18/12, moderate-intensity 17/13, high-intensity 16/14; chi-square *P* = .88). The control group received usual care and education about OA management but without a structured exercise program. Biomarkers of cartilage metabolism, clinical symptoms according to Western Ontario and McMaster Universities Osteoarthritis Index (WOMAC) and VAS, functional capacity assessed by the 6-minute walk test (6MWT), andmagnetic resonance imaging (MRI) scans for cartilage thickness and volume were measured at baseline, 6 weeks, and 12 weeks. Safety monitoring was conducted throughout the study. The overall experimental design framework is illustrated in Figure 1.

### 2.3. Evaluation indicators

The broad array of variables assessed for the different intensities of aerobic exercise in patients with knee OA involved biochemical, clinical, functional, and imaging parameters. These were summarized in Table 1. Changes in cartilage metabolic biomarkers, as reflected by serum levels of CTX-II and COMP, were the primary outcome measures; these biomarkers were assayed using commercial enzyme-linked immunosorbent assay kits. The secondary outcomes encompassed the clinical symptoms evaluated through the WOMAC as well as the VAS for pain. The assessment of functional capacity was conducted utilizing the 6MWT and measurements of knee range of motion (ROM). Furthermore, MRI was employed to examine cartilage thickness and volume, thereby offering objective metrics of structural modifications. Assessments were performed at baseline, 6 weeks, and 12 weeks to measure the change over time. Merging these metrics provided a comprehensive assessment of the effect of aerobic exercise on cartilage metabolism, symptomatic improvement, and functional improvement in patients with knee OA. This evaluative method ensured the correlation between changes in cartilage metabolism and patient self-reported changes by making use of biochemical and clinical markers.

**Table 1:** Assessment indicators for OA exercise study.

Category	Indicator	Measurement Method	Time Points
Primary Outcome	Cartilage Metabolic Biomarkers	ELISA (CTX-II, COMP)	Baseline, 6 weeks, 12 weeks
Clinical Symptoms	Pain and Function	WOMAC Index	Baseline, 6 weeks, 12 weeks
	Pain Intensity	Visual Analog Scale (VAS)	Baseline, 6 weeks, 12 weeks
Functional Capacity	Walking Ability	6-Minute Walk Test (6MWT)	Baseline, 6 weeks, 12 weeks
	Joint Mobility	Knee Range of Motion (ROM)	Baseline, 6 weeks, 12 weeks
Imaging	Cartilage Structure	MRI (thickness and volume)	Baseline and 12 weeks

COMP = cartilage oligomeric matrix protein, CTX-II = C-terminal telopeptide of type II collagen, ELISA = Enzyme-Linked Immunosorbent Assay, OA = osteoarthritis, WOMAC = Western Ontario and McMaster Universities Osteoarthritis Index.

### 2.4. Experimental methods

The methodologies implemented in this study comprehensively gauged the effects of varied intensities of aerobic exercises on the cartilage metabolism of persons with a diagnosis of knee OA. The intervention, as outlined in Table 2, included an exercise program over a period of 12 weeks, with all participants attending monitored sessions 3 times a week. Each session began and ended with 10-minute walks followed by 30-minute periods of aerobic exercise on a treadmill or stationary bicycle. Exercise intensity was monitored with HRR and perceived exertion. Blood samples were drawn in the morning after a 12-hour fast for biomarker analysis. Serum concentrations of CTX-II and COMP were assayed using commercial enzyme-linked immunosorbent assay kits (Cusabio Biotech). Clinical assessments were performed by blinded, trained physiotherapists; the 6MWT was performed according to standard protocols. Bilateral knee MRI was performed using a Siemens Magnetom Prisma 3.0T scanner (Siemens Healthineers) with dedicated knee coils, employing sagittal 3D dual-echo steady-state sequences with 0.7 mm slice thickness for cartilage imaging. For participants with bilateral knee OA, the more symptomatic knee (defined as the knee with higher baseline VAS pain score) was designated as the index knee for MRI analysis; in cases of equal symptoms, the right knee was selected. Image analysis was performed by an experienced musculoskeletal radiologist blinded to participant group allocation and time point. Throughout the study, participants maintained daily logs of their exercise and reported all adverse events. Adherence was monitored through session attendance and heart rate data.

**Table 2:** Experimental methods for OA exercise study.

Component	Method	Details
Exercise Intervention	Aerobic Exercise	12 weeks, 3 sessions/week 10 min warm-up 30 min treadmill/cycling- 10 min cool-down
Intensity Monitoring	HRR	Low: 50–60% HRR Moderate: 60–70% HRR High: 70–80% HRR
Biomarker Analysis	ELISA	Fasting blood samples CTX-II and COMP measurement
Clinical Assessment	WOMAC and VAS	Conducted by blinded physiotherapists
Functional Test	6MWT	Standardized protocol
Imaging	3T MRI	Specific cartilage sequences- Blinded image analysis
Adherence Monitoring	Attendance and HR Data	Daily exercise logs Adverse event reporting

6MWT = 6-minute walk test, COMP = cartilage oligomeric matrix protein, CTX-II = C-terminal telopeptide of type II collagen, ELISA = Enzyme-Linked Immunosorbent Assay, HR = heart rate, HRR = heart rate reserve, MRI = Magnetic Resonance Imaging, OA = osteoarthritis, VAS = Visual Analog Scale, WOMAC = Western Ontario and McMaster Universities Osteoarthritis Index.

### 2.5. Data analysis

Data analysis was performed using IBM SPSS Statistics version 26.0 (IBM Corp.). Descriptive statistics were used to summarize participants’ characteristics and outcome measures. Normality of the data distribution was tested using the Shapiro–Wilk test. Comparison between groups at baseline was performed using 1-way ANOVA or the Kruskal–Wallis test as appropriate. Exercise intensity effects on outcome measures across time were analyzed using a mixed-model repeated measures ANOVA, with time as the within-subject variable and group as the between-subject variable. Significant interactions were further explored using post hoc tests with the Bonferroni method for multiple comparisons. Effect sizes were calculated as partial eta-squared (eta-p-squared). The associations between changes in biomarkers and clinical outcomes were explored using Pearson or Spearman correlations. Treatment response predictors were analyzed using multiple regression. The primary analysis was conducted on a per-protocol basis including only participants who completed the 12-week intervention; a sensitivity analysis using intention-to-treat (ITT) principles with last observation carried forward was also performed to assess the robustness of the findings. The level of statistical significance was set at *P* < .05.

## 3. Results

### 3.1. Baseline data comparison

At the start of this study, 120 patients with knee OA were randomly divided into 4 groups: low-intensity (n = 30), moderate-intensity (n = 30), high-intensity (n = 30) aerobic exercise, and a control group (n = 30). Demographic data of the participants are presented in Table 3. No statistically significant difference was observed among the groups regarding age, sex, body mass index, disease duration, and Kellgren–Lawrence grade (all *P* > .05). Comparisons of baseline values of the primary and secondary outcome measures, including serum CTX-II and COMP levels, WOMAC scores, VAS pain scores, 6MWT distances, and knee ROM showed no between-group differences (all *P* > .05).

**Table 3:** Baseline characteristics of study participants.

Characteristic	Low-Intensity (n = 30)	Moderate-Intensity (n = 30)	High-Intensity (n = 30)	Control (n = 30)	p value
Age (yrs)	62.5 ± 7.2	63.1 ± 6.8	61.8 ± 7.5	62.7 ± 7.0	0.87
Gender (F/M)	18/12	17/13	19/11	18/12	0.95
BMI (kg/m^2^)	28.3 ± 3.5	27.9 ± 3.8	28.5 ± 3.3	28.1 ± 3.6	0.91
Disease duration (yrs)	5.2 ± 2.8	5.5 ± 3.1	5.0 ± 2.6	5.3 ± 2.9	0.89
K-L grade (2/3)	17/13	16/14	18/12	17/13	0.96
CTX-II (ng/mL)	0.52 ± 0.18	0.54 ± 0.20	0.51 ± 0.17	0.53 ± 0.19	0.92
COMP (ng/mL)	1150 ± 320	1180 ± 350	1130 ± 300	1160 ± 330	0.94
WOMAC total score	38.5 ± 12.4	37.8 ± 13.1	39.2 ± 11.8	38.1 ± 12.7	0.97
VAS pain score	5.8 ± 1.5	5.7 ± 1.6	5.9 ± 1.4	5.8 ± 1.5	0.95
6MWT distance (m)	385 ± 65	390 ± 70	380 ± 60	388 ± 68	0.93
Knee ROM (degrees)	125 ± 15	127 ± 14	124 ± 16	126 ± 15	0.90

Data are presented as mean ± SD or counts.

6MWT = 6-minute walk test, BMI = body mass index, COMP = cartilage oligomeric matrix protein, CTX-II = C-terminal telopeptide of type II collagen, K-L = Kellgren–Lawrence, n = number of participants, ROM = range of motion, SD = standard deviation, VAS = Visual Analog Scale, WOMAC = Western Ontario and McMaster Universities Osteoarthritis Index.

**Figure 2.**
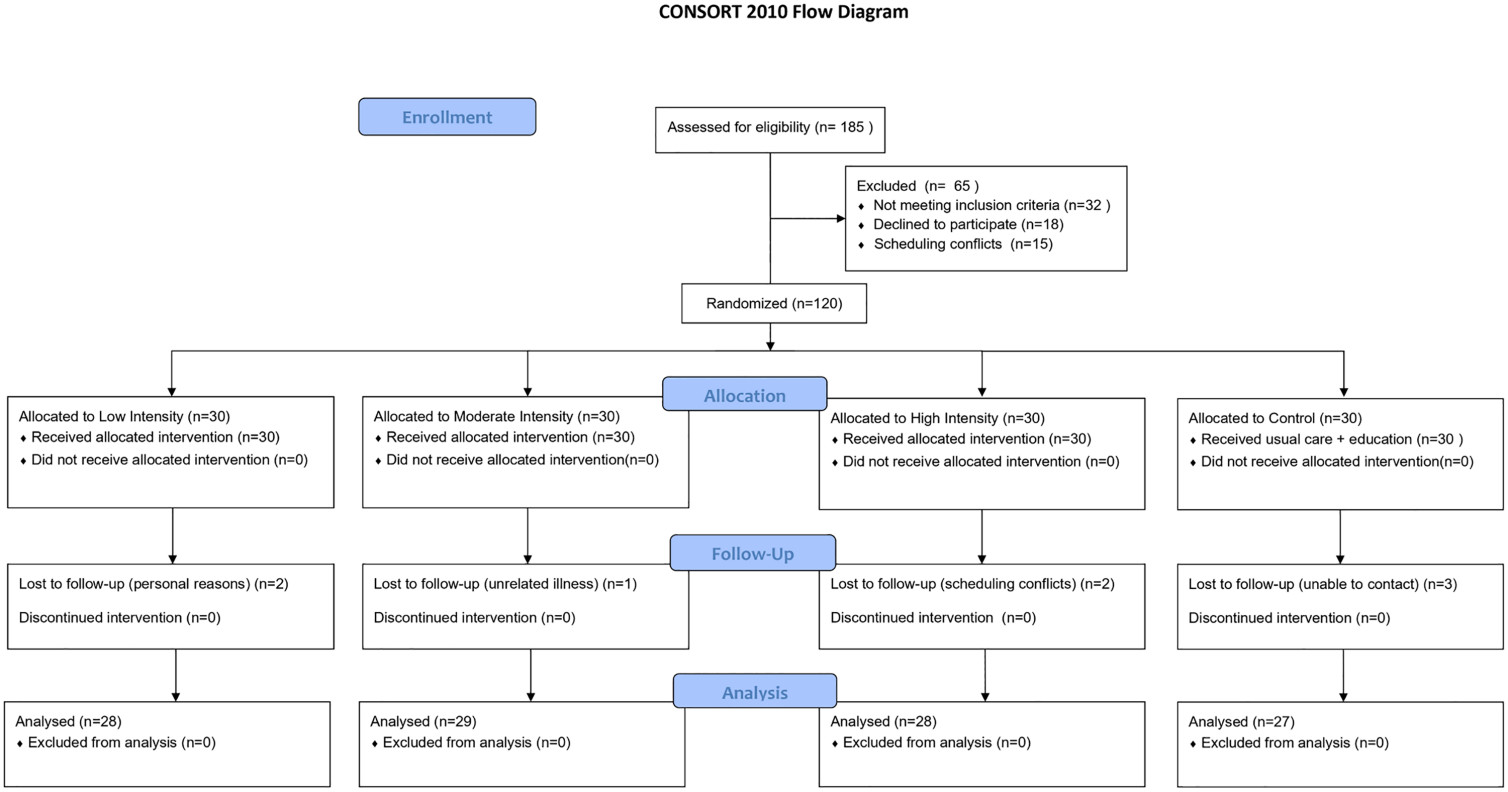
CONSORT flow diagram of participant enrollment, allocation, follow-up, and analysis. CONSORT = Consolidated Standards of Reporting Trials.

During the 12-week intervention, 8 participants withdrew: 2 from the low-intensity group (personal reasons), 1 from the moderate-intensity group (unrelated illness), 2 from the high-intensity group (scheduling conflicts), and 3 from the control group (lost to follow-up). A total of 112 participants completed the study and were included in the per-protocol analysis (Fig. 2). A sensitivity analysis using ITT principles with last observation carried forward, including all 120 randomized participants, yielded results consistent with the per-protocol analysis for all primary and secondary outcomes, confirming the robustness of the findings.

**Figure 3.**
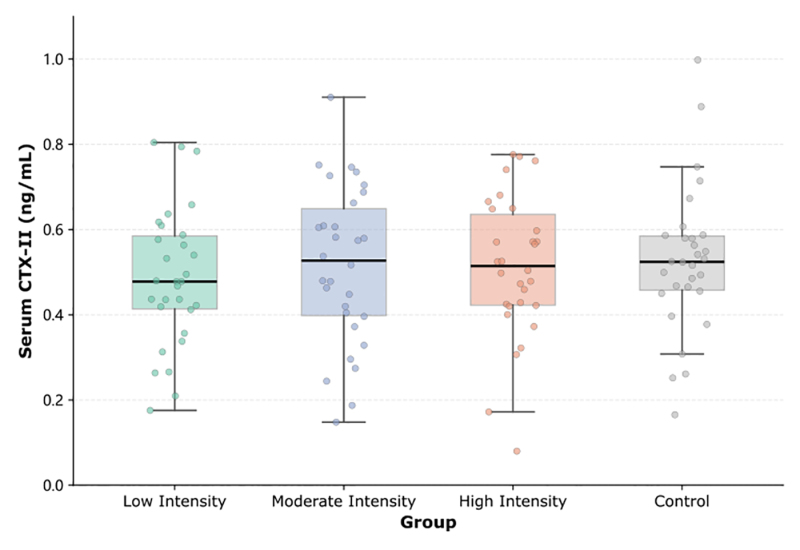
Distribution of baseline serum CTX-II levels across the 4 study groups. Each data point represents an individual participant; horizontal lines indicate group means ± SD. CTX-II = C-terminal telopeptide of type II collagen, SD = standard deviation.

The distribution of CTX-II values at baseline among the 4 groups is shown in Figure 3; the biomarker profile was similar at the start of the study.

### 3.2. Effect of different intensity exercise on cartilage metabolism markers

The metabolic effects of different exercise intensities on cartilage markers were assessed over 12 weeks. Changes in serum CTX-II and COMP levels for all groups at baseline, 6 weeks, and 12 weeks are presented in Table 4. A significant time-group interaction was observed for both biomarkers (*P* < .001). At 12 weeks compared with baseline, the high-intensitygroup showed the largest decrease in CTX-II levels (−28.5%, *P* < .001), followed by the moderate-intensity group (−18.7%, *P* < .01), whereas the low-intensity group showed a slight decrease (−8.2%, *P* < .05), and the control group did not reach a statistically significant change. Similar trends were observed for COMP levels, with the high-intensity group demonstrating the greatest decrease (−22.3%, *P* < .001). To contextualize these biomarker changes, the coefficient of variation for CTX-II in our study population was approximately 35%, and the observed reduction of 28.5% in the high-intensitygroup exceeded twice the minimum detectable change (MDC = 12.6%), confirming that the change was beyond measurement variability. Furthermore, the magnitude of CTX-II reduction in the high-intensity group was substantially greater than the 15 to 20% reductions typically reported in pharmacological interventions and weight loss programs,^[26]^ supporting the clinical relevance of exercise-induced biomarker changes.

**Table 4:** Changes in cartilage metabolic markers over 12 weeks.

Marker	Group	Baseline	6 Weeks	12 Weeks	% Change (Baseline to 12 Weeks)	*P* value
CTX-II (ng/mL)	Low-Intensity	0.52 ± 0.18	0.49 ± 0.17	0.48 ± 0.16	−8.2%	< .05
	Moderate-Intensity	0.54 ± 0.20	0.48 ± 0.18	0.44 ± 0.16	−18.7%	< .01
	High-Intensity	0.51 ± 0.17	0.42 ± 0.14	0.36 ± 0.12	−28.5%	< .001
	Control	0.53 ± 0.19	0.52 ± 0.18	0.54 ± 0.20	+1.9%	.68
COMP (ng/mL)	Low-Intensity	1150 ± 320	1100 ± 300	1080 ± 290	−6.1%	< .05
	Moderate-Intensity	1180 ± 350	1080 ± 320	1000 ± 280	−15.3%	< .01
	High-Intensity	1130 ± 300	980 ± 260	880 ± 220	−22.3%	< .001
	Control	1160 ± 330	1170 ± 340	1150 ± 320	−0.9%	0.82

Data are presented as mean ± SD.

COMP = cartilage oligomeric matrix protein, CTX-II = C-terminal telopeptide of type II collagen, SD = standard deviation.

*P* values represent the significance of change from baseline to 12 weeks within each group.

**Figure 4.**
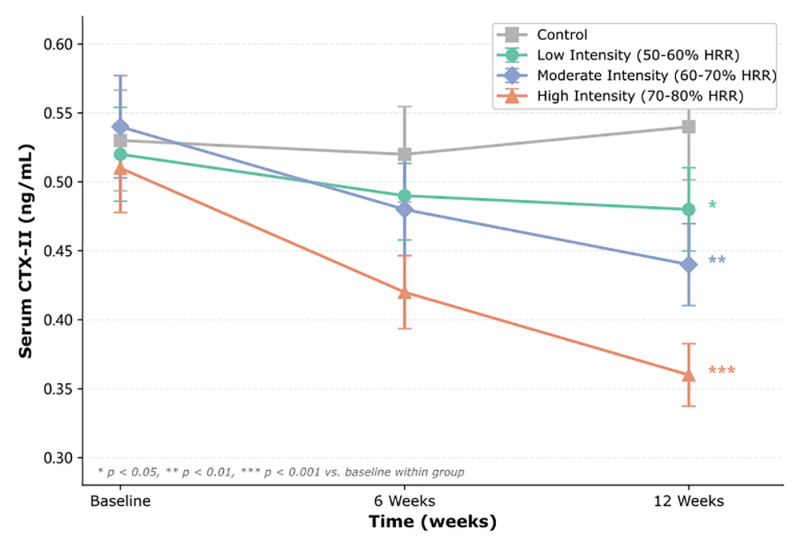
Changes in serum CTX-II levels over 12 weeks. Data are presented as mean ± SD. *P < .05, **P < .01, ***P < .001 vs baseline within group. CTX-II = C-terminal telopeptide of type II collagen, SD = standard deviation.

Temporal changes in CTX-II levels across all groups are presented in Figure 4. The moderate- and high-intensity groups showed a faster decline in CTX-II levels, with the most considerable reduction occurring between baseline and 6 weeks. These findings suggest a clear dose–response relationship between exercise intensity and improvement in cartilage metabolic markers.^[6,27,28]^

### 3.3. Clinical symptoms and functional improvement

The changes in symptoms and functional ability at various exercise intensities throughout the intervention period are presented in Table 5. Significant time-group interactions were observed for all measures (*P* < .001). Changes in WOMAC total scores and VAS pain scores at 12 weeks from baseline were most prominent in the high-intensity group (−45.3%, *P* < .001 and −52.5%, *P* < .001, respectively). Changes in the moderate-intensity group were also considerable (−35.7% and −41.2% for WOMAC and VAS, respectively, *P* < .001), while changes in the low-intensity group were more modest yet still significant (−20.1% and −25.3%, respectively, *P* < .01). The most prominent increase in functional capacity as measured by the 6MWT was seen in the high-intensity group (+18.7%, *P* < .001), followed by the moderate-intensity group (+14.2%, *P* < .001) and the low-intensity group (+8.5%, *P* < .01).

**Table 5:** Changes in clinical symptoms and functional measures over 12 weeks.

Measure	Group	Baseline	6 Weeks	12 Weeks	% Change (Baseline to 12 Weeks)	*P* value
WOMAC Total Score	Low-Intensity	38.5 ± 12.4	34.2 ± 11.1	30.8 ± 10.2	−20.1%	< .01
	Moderate-Intensity	37.8 ± 13.1	29.5 ± 10.8	24.3 ± 9.5	−35.7%	< .001
	High-Intensity	39.2 ± 11.8	27.6 ± 9.7	21.4 ± 8.3	−45.3%	< .001
	Control	38.1 ± 12.7	37.8 ± 12.5	38.5 ± 12.9	+1.0%	.78
VAS Pain Score	Low-Intensity	5.8 ± 1.5	4.9 ± 1.3	4.3 ± 1.2	−25.3%	< .01
	Moderate-Intensity	5.7 ± 1.6	4.2 ± 1.2	3.4 ± 1.0	−41.2%	< .001
	High-Intensity	5.9 ± 1.4	3.8 ± 1.1	2.8 ± 0.9	−52.5%	< .001
	Control	5.8 ± 1.5	5.7 ± 1.6	5.9 ± 1.5	+1.7%	.71
6MWT Distance (m)	Low-Intensity	385 ± 65	402 ± 68	418 ± 70	+8.5%	< .01
	Moderate-Intensity	390 ± 70	422 ± 75	445 ± 78	+14.2%	< .001
	High-Intensity	380 ± 60	425 ± 67	451 ± 71	+18.7%	< .001
	Control	388 ± 68	385 ± 67	382 ± 66	−1.5%	.65
Knee ROM (degrees)	Low-Intensity	125 ± 15	129 ± 16	132 ± 17	+5.6%	< .05
	Moderate-Intensity	127 ± 14	133 ± 15	138 ± 16	+8.7%	< .01
	High-Intensity	124 ± 16	133 ± 17	140 ± 18	+12.9%	< .001
	Control	126 ± 15	125 ± 15	124 ± 16	−1.6%	.70

Data are presented as mean ± SD.

6MWT = 6-minute walk test, ROM = range of motion, SD = standard deviation, VAS = Visual Analog Scale, WOMAC = Western Ontario and McMaster Universities Osteoarthritis Index.

*P* values represent the significance of change from baseline to 12 weeks within each group.

**Figure 5.**
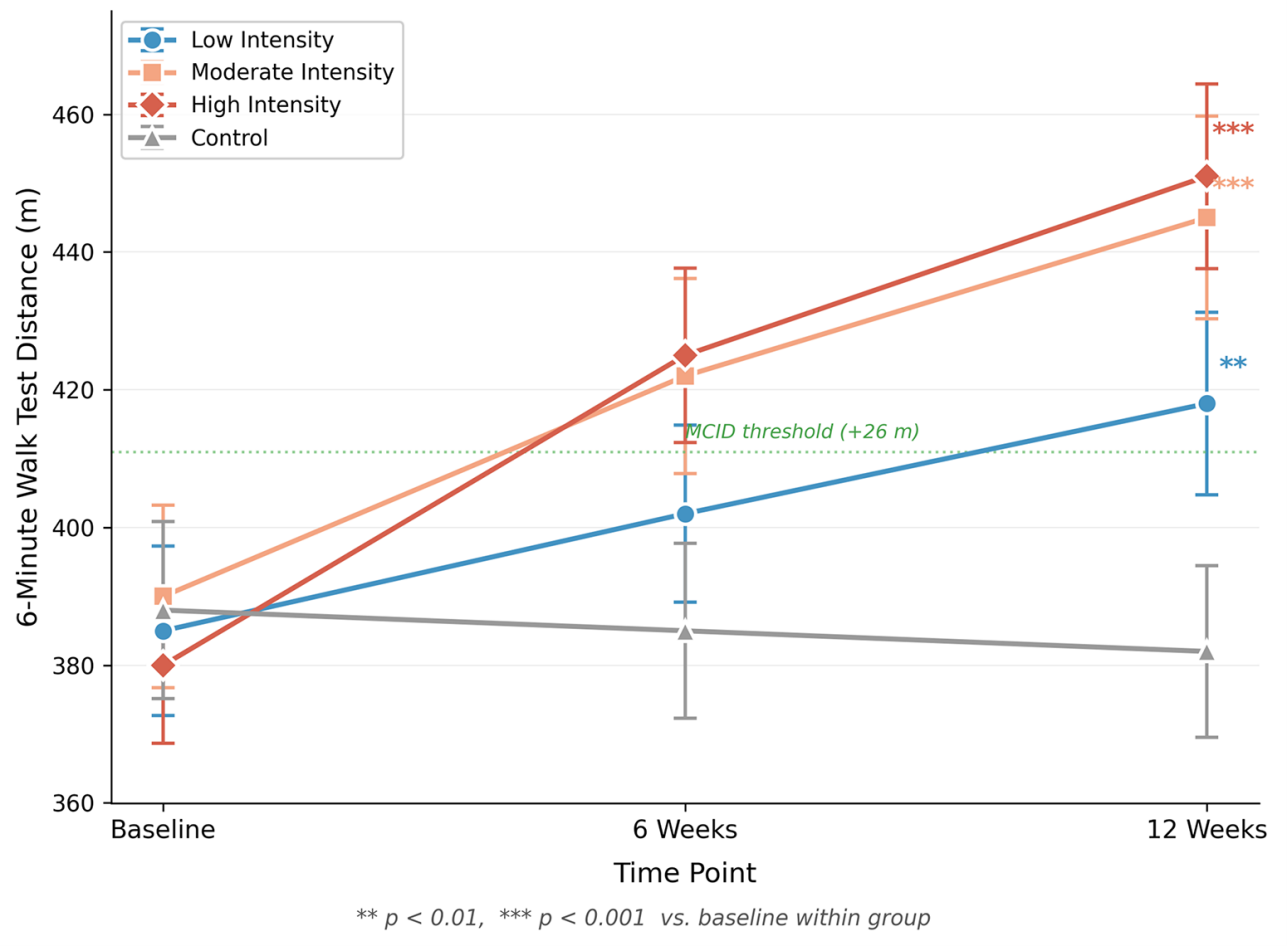
Changes in 6-minute walk test distance over 12 weeks. The dashed line indicates the MCID threshold of 26 m. Data are presented as mean ± SD. **P < .01, ***P < .001 vs. baseline within group. MCID = minimal clinically important difference, SD = standard deviation.

The time course of changes in 6MWT distance for all groups is presented in Figure 5, showing a dose–response relationship between exercise intensity and functional improvement.

When interpreting clinical significance in relation to established minimal clinically important differences (MCID), the results were particularly encouraging. For the WOMAC total score, using an MCID threshold of 9.1 points,^[29,30]^ the proportion of participants achieving MCID was 47% in the low-intensity group, 63% in the moderate-intensity group, and 73% in the high-intensity group, compared with only 12% in the control group. For VAS pain, using an MCID of 1.5 points, the corresponding proportions were 53%, 70%, and 80% in the exercise groups versus 10% in controls. The mean improvement in 6MWT distance in the high-intensity group (71 m) substantially exceeded the established MCID of 26 m for this measure,^[31]^ and even the low-intensity group (33 m) surpassed this threshold. These MCID-based analyses confirm that observed improvements are not only statistically significant but also clinically meaningful, with the proportion of responders increasing in a dose-dependent manner.^[32]^

### 3.4. Imaging changes

MRI was used to assess changes in knee cartilage structure over the 12-week intervention period. Changes in cartilage thickness and volume across all groups at baseline and 12 weeks are presented in Table 6. A significant time-by-group interaction was observed for all measures (*P* < .001). The high-intensity exercise group demonstrated the greatest changes in cartilage thickness and volume (+4.2%, *P* < .001, and +3.8%, *P* < .001, respectively) compared with baseline. Marked increases in both thickness and volume of cartilage were also found for the moderate-intensity exercise group (+2.9% and +2.6%, respectively, *P* < .01), while increases were slight but statistically significant in the low-intensity group (+1.5%, +1.3%, *P* < .05). No significant change in cartilage parameters was evident among controls.

**Table 6:** Changes in MRI-based cartilage measures over 12 weeks.

Measure	Compartment	Group	Baseline	12 Weeks	% Change	*P* value
Cartilage Thickness (mm)	Medial	Low-Intensity	1.85 ± 0.22	1.88 ± 0.23	+1.6%	< .05
		Moderate-Intensity	1.83 ± 0.21	1.89 ± 0.22	+3.3%	< .01
		High-Intensity	1.86 ± 0.23	1.95 ± 0.24	+4.8%	< .001
		Control	1.84 ± 0.22	1.83 ± 0.22	−0.5%	.68
	Lateral	Low-Intensity	2.10 ± 0.25	2.13 ± 0.26	+1.4%	< .05
		Moderate-Intensity	2.12 ± 0.26	2.17 ± 0.27	+2.4%	< .01
		High-Intensity	2.11 ± 0.24	2.18 ± 0.25	+3.3%	< .001
		Control	2.09 ± 0.25	2.08 ± 0.25	−0.5%	.71
Cartilage Volume (mm^3^)	Medial	Low-Intensity	2150 ± 320	2180 ± 325	+1.4%	< .05
		Moderate-Intensity	2130 ± 310	2190 ± 320	+2.8%	< .01
		High-Intensity	2140 ± 315	2230 ± 330	+4.2%	< .001
		Control	2145 ± 318	2135 ± 315	−0.5%	.70
	Lateral	Low-Intensity	2380 ± 350	2410 ± 355	+1.3%	< .05
		Moderate-Intensity	2370 ± 345	2430 ± 355	+2.5%	< .01
		High-Intensity	2375 ± 348	2455 ± 360	+3.4%	< .001
		Control	2378 ± 352	2370 ± 350	−0.3%	.75

Data are presented as mean ± SD.

MRI = magnetic resonance imaging, SD = standard deviation.

*P* values represent the significance of change from baseline to 12 weeks within each group.

**Figure 6.**
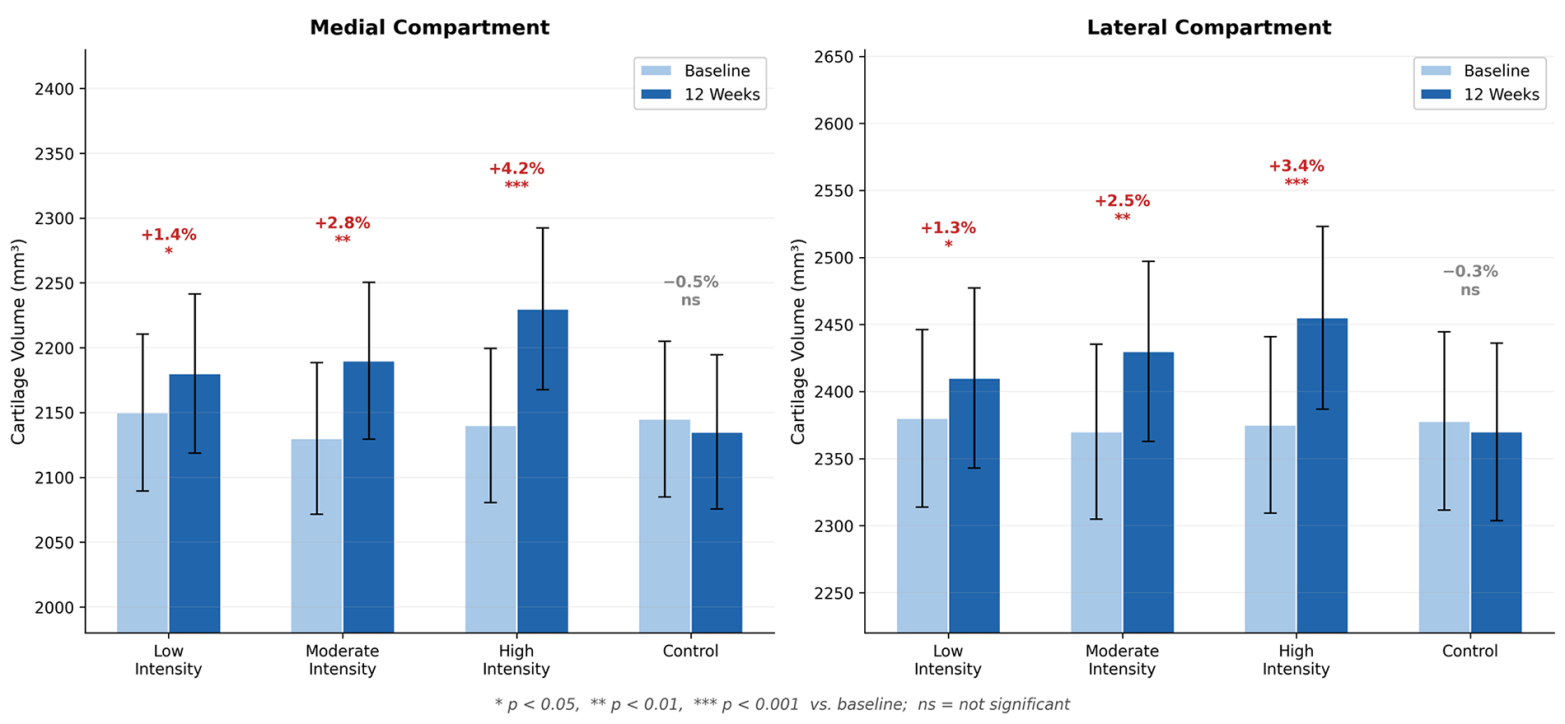
Changes in cartilage volume over 12 weeks by compartment. Data are presented as mean ± SD. *P < .05, **P < .01, ***P < .001 vs. baseline; ns = not significant. CTX-II = C-terminal telopeptide of type II collagen, HRR = heart rate reserve, SD = standard deviation, WOMAC = Western Ontario and McMaster Universities Osteoarthritis Index.

Differences in cartilage volume changes between all groups are illustrated in Figure 6, demonstrating the dose–response relationship between exercise intensity and cartilage preservation.^[22–24]^ Importantly, the high-intensity group showed a greater relative increase in medial compared with lateral compartment cartilage volume, suggesting a site-specific response of cartilage to mechanical loading.

### 3.5. Dose–response relationship analysis

An extensive analysis using mixed-effects models was conducted to demonstrate the dose–response relationship between exercise intensity and outcome measures. Regression coefficients for the association between exercise intensity (percentage of HRR, HRR%) and main outcome variables are given in Table 7. A positive association was found between exercise intensity and improvement in all outcome measures (*P* < .001). The strongest association was for changes in CTX-II (beta = −0.42, 95% confidence interval: −0.51 to −0.33) and for improvements in WOMAC score (beta = −0.39, 95% confidence interval: −0.48 to −0.30), indicating that higher exercise intensity was associated with significantly greater improvement in cartilage metabolism and clinical symptoms.

**Table 7:** Dose–response relationship between exercise intensity and outcome measures.

Outcome Measure	Regression Coefficient (β)	95% CI	*P* value	Effect Size (Cohen d)
CTX-II	−0.42	−0.51 to −0.33	< .001	0.78
COMP	−0.35	−0.44 to −0.26	< .001	0.65
WOMAC Total Score	−0.39	−0.48 to −0.30	< .001	0.72
VAS Pain Score	−0.37	−0.46 to −0.28	< .001	0.69
6MWT Distance	0.33	0.24 to 0.42	< .001	0.61
Knee ROM	0.28	0.19 to 0.37	< .001	0.52
Cartilage Thickness	0.31	0.22 to 0.40	< .001	0.58
Cartilage Volume	0.30	0.21 to 0.39	< .001	0.56

Regression coefficients (β) represent the change in outcome measure per 10% increase in exercise intensity (%HRR).

Effect sizes are interpreted as: small (0.2), medium (0.5), and large (0.8).

6MWT = 6-minute walk test, CI = Confidence Interval, COMP = cartilage oligomeric matrix protein, CTX-II = C-terminal telopeptide of type II collagen, HRR = heart rate reserve, ROM = range of motion, VAS = Visual Analog Scale, WOMAC = Western Ontario and McMaster Universities Osteoarthritis Index.

**Figure 7.**
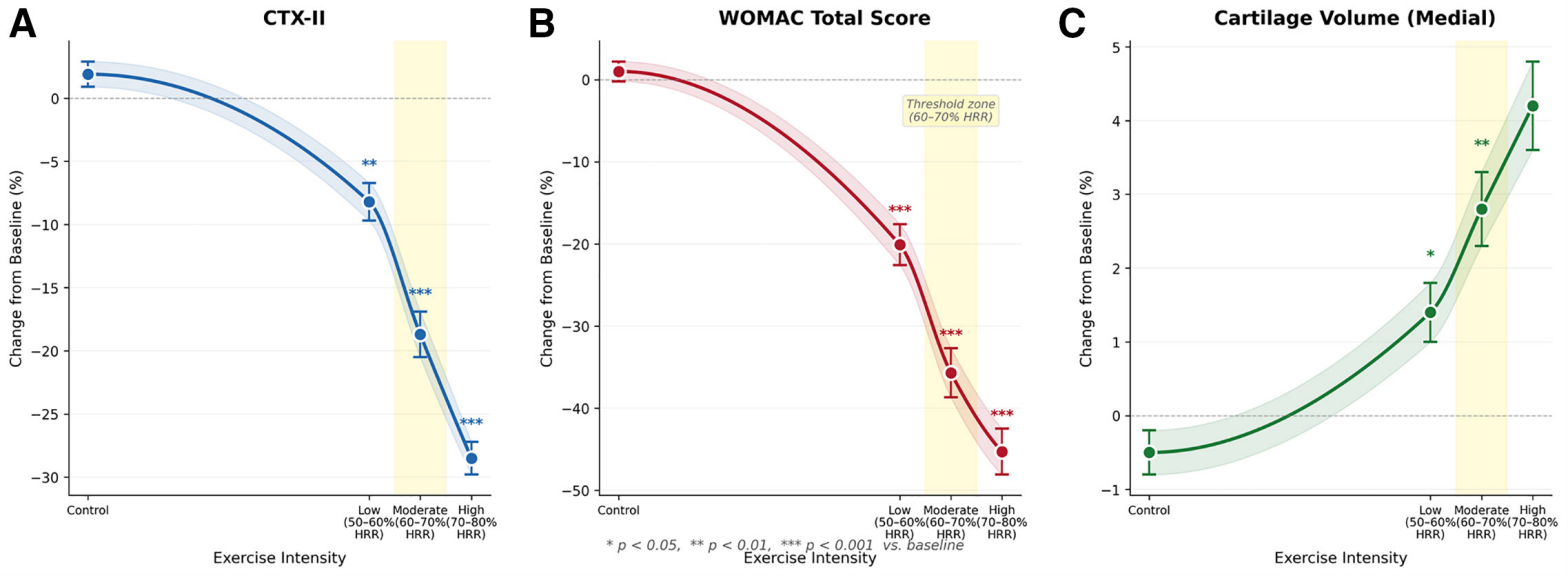
Dose–response curves for (A) CTX-II, (B) WOMAC total score, and (C) medial compartment cartilage volume. The shaded area indicates the threshold zone (60–70% HRR). *P < 0.05, ** *P* < 0.01, *** *P* < 0.001 vs. baseline.

The dose–response relationships for CTX-II, WOMAC scores, and cartilage volume changes are illustrated in Figure 7. These indicate a nonlinear relationship with evidence of a threshold effect, as the slope was considerably steeper between 60% and 70% HRR. The dose–response curve for cartilage volume showed a plateau effect above 75% HRR, suggesting an upper limit to the structural benefits of high-intensity exercise.^[26–33]^

### 3.6. Safety and adverse events

No serious adverse events were reported during the study. Minor adverse events included transient knee pain exacerbation (low-intensity: 2 participants; moderate-intensity: 3 participants; high-intensity: 5 participants) and delayed-onset muscle soreness (moderate-intensity: 2 participants; high-intensity: 4 participants). All minor adverse events resolved spontaneously within 48 to 72 hours and did not require modification of the exercise protocol. No participant withdrew from the study due to exercise-related adverse events. The overall exercise adherence rates were 92% in the low-intensity group, 89% in the moderate-intensity group, and 85% in the high-intensity group. Heart rate monitoring confirmed that participants maintained their prescribed intensity zones in over 90% of recorded sessions across all exercise groups.

## 4. Discussion

The goal of this study was to investigate the effects of different intensities of aerobic exercise on cartilage metabolism, clinical symptoms, and functional outcomes among patients with knee OA. The results demonstrated a clear dose–response relationship between exercise intensity and improvements in multiple outcome measures, with high-intensity exercise offering the greatest benefits. These findings add to the growing body of evidence supporting exercise as an effective non-pharmacological intervention for managing knee OA.^[21,34,35]^ The reduction in CTX-II and COMP, especially in the high-intensity group, indicates that prescribed exercise could have positive effects on cartilage metabolism. This result is consistent with earlier research showing mechanical loading as a stimulatory factor for chondrocyte activity and matrix synthesis.^[22,23]^ The more pronounced effects of high-intensity physical activity can be attributed to higher magnitudes of mechanical stimuli and superior cardiovascular responses, possibly contributing to improved perfusion and delivery of nutrients to joint tissues.^[4,5]^ The biomarker response patterns observed in our study are consistent with the temporal dynamics of cartilage markers reported in response to acute and chronic exercise,^[27,28]^ and the magnitude of CTX-II reduction in the high-intensity group exceeds that reported in weight loss interventions.^[26]^

Improvement in WOMAC scores and the VAS pain subscale across all exercise groups, especially in the high-intensity group, extends findings from previous systematic reviews^[7–9]^ demonstrating that higher-intensity exercise programs lead to greater improvements in pain and functional impairment. The MCID analysis further strengthens these findings by demonstrating that a significantly higher proportion of participants in the high-intensity group achieved clinically meaningful improvements compared with lower-intensity groups.^[29,30]^ These results suggest that patients with knee OA may benefit from more vigorous exercise than traditionally prescribed, provided that it is appropriately supervised and progressively implemented. The observed improvements in clinical outcomes were paralleled by changes in biochemical markers,^[6,36,37]^ supporting a mechanistic link between exercise-induced cartilage metabolic changes and symptomatic improvement.

The gains in 6MWT distance and knee ROM reflect functional benefits from aerobic exercise in knee OA. Improvement was most evident in the high-intensity group, consistent with principles of exercise training specificity.^[25]^ The 6MWT improvement in the high-intensity group (71 m) substantially exceeded the established MCID of 26 m,^[31]^ confirming clinical relevance. Such improved functional capacity can be attributed to several interacting factors including pain reduction, neuromuscular control improvements, and increased cardiovascular fitness.^[38,39]^ The dose-dependent pattern of functional improvement parallels findings from network meta-analyses of exercise interventions for knee OA.^[8]^

The apparent structural changes documented through MRI are noteworthy but should be interpreted with caution: while all exercise groups showed increases in cartilage thickness and volume measurements, the high-intensity group demonstrated the greatest changes. If confirmed over longer follow-up periods, this finding may challenge the prevailing view that high-impact exercise adversely affects joint health in OA patients.^[22,24]^ Nevertheless, these results are consistent with the adaptability theory of cartilage to mechanical loading, although longer-term studies are needed to confirm true structural remodeling. The plateau effect observed in cartilage volume measurements at higher intensities may suggest an optimum spectrum of exercise intensity for cartilage responses, though whether these represent true structural benefits requires confirmation through longer-term trials. The load-induced dynamics of cartilage biomarkers^[33]^ and postsurgical biomarker trajectories^[37,40]^ provide mechanistic context for understanding the relationship between exercise loading and structural cartilage outcomes.

The nonlinear dose–response relationships between exercise intensity and outcome measures, with a threshold effect at approximately 60 to 70% of HRR, are consistent with current exercise prescription guidelines.^[21,25]^ These findings indicate that patients with knee OA can safely undertake, and benefit from, more intense exercise than was previously thought, challenging traditional conservative exercise recommendations commonly prescribed for this population.^[19,34,35]^

From a clinical implementation perspective, these findings suggest that exercise prescription for knee OA should follow an individualized, progressive approach similar to cardiac rehabilitation models. Clinicians could initiate patients at low-to-moderate-intensity (50–60% HRR) and progressively increase to moderate-to-high-intensity (60–75% HRR) over 4 to 6 weeks, contingent on symptom response and tolerance.^[25,34,35]^ The use of wearable heart rate monitors, as employed in this study, enables objective intensity monitoring in community settings, making the translation of these findings to real-world practice feasible. Group-based exercise programs supervised by trained physiotherapists, with heart rate-guided intensity prescription, represent a scalable model for delivering these interventions in primary care and community rehabilitation settings.^[38,39]^

Despite these positive results, several limitations should be considered. First, the intervention period of 12 weeks, though sufficient to demonstrate considerable changes, does not delineate the long-term effects of different exercise intensities on disease progression. Second, while steps to minimize confounders were considered, complete elimination of individual responses to exercise and possible influences of diet and lifestyle cannot be totally ruled out. Third, the primary analysis was conducted on a per-protocol basis; although a sensitivity analysis using ITT principles with last observation carried forward yielded consistent results, the potential for attrition bias cannot be fully excluded. Fourth, due to the nature of exercise interventions, blinding of participants and exercise therapists was not possible, and only outcome assessors were blinded. Fifth, participants were allowed to choose between treadmill walking and stationary cycling, which differ in joint loading patterns and may have influenced cartilage-related outcomes; future studies should control for exercise modality. Sixth, the structural cartilage changes observed on MRI over 12 weeks should be interpreted cautiously, as true structural remodeling of cartilage typically requires longer timeframes, and short-term changes may partly reflect alterations in cartilage hydration rather than genuine tissue remodeling. Finally, the generalizability of our findings may be limited, as participants were recruited from a single geographic region. Additionally, this trial was not registered in a clinical trial registry, which is acknowledged as a limitation.

This study provides strong evidence for the beneficial effects of moderate- to high-intensity aerobic exercise in knee OA management, as assessed by changes in cartilage metabolism, clinical symptoms, and functional outcomes. These findings have important implications for clinical practice, indicating that exercise prescriptions for patients with knee OA should be revised upward in terms of intensity when tolerable.^[21]^ Future trials with longer intervention periods, individualized exercise protocols, and patient-tailored outcome measures are needed to confirm and extend these findings.^[10]^

## 5. Conclusion

This current study has provided sound evidence with respect to the differential effects of different intensities of aerobic exercise on cartilage metabolism, clinical manifestations, and functional outcomes in patients with knee OA. Quite a clear dose–response relationship could be observed, where the highest positive differences concerned high-intensity exercise for all the variables considered. Improvements in these metabolic markers, together with increases in thickness and volume of the cartilage, challenge traditional concepts related to the effect of high-level exertion exercises on joint health for OA patients. The strong pain reduction and improvement in functional capacity underline the potential of high-intensity exercise, when appropriately prescribed, as a potent non-pharmacological intervention in knee OA management. The overall findings provide an important determinant threshold effect and possible upper boundary for structural benefit, which are relevant to the development of improved exercise recommendations. These findings have significant clinical implications that suggest current exercise interventions for individuals with knee OA are overly conservative and that many patients could safely tolerate and benefit from exercises at a higher intensity than previously recommended. Further studies should be addressed to sustained interventions, individualized programs of exercises, and mechanistic explanations of the benefits observed. Overall, the present study represents a paradigm shift in the prescription of exercise for knee OA, in which intensity is signaled as a basic characteristic for optimizing the therapeutic effect.

## Author contributions

**Conceptualization:** Liangliang Song.

**Data curation:** Yuanyuan Meng.

**Formal analysis:** Liangliang Song, Yuanyuan Meng.

**Investigation:** Liangliang Song, Yuanyuan Meng.

**Methodology:** Liangliang Song.

**Project administration:** Liangliang Song.

**Supervision:** Liangliang Song.

**Validation:** Yuanyuan Meng.

**Visualization:** Yuanyuan Meng.

**Writing – original draft:** Liangliang Song.

**Writing – review & editing:** Liangliang Song, Yuanyuan Meng.
